# Altered Amygdala Resting-State Functional Connectivity and Hemispheric Asymmetry in Patients With Social Anxiety Disorder

**DOI:** 10.3389/fpsyt.2018.00164

**Published:** 2018-04-26

**Authors:** Ye-Ha Jung, Jung E. Shin, Yoonji I. Lee, Joon H. Jang, Hang J. Jo, Soo-Hee Choi

**Affiliations:** ^1^Department of Psychiatry, Seoul National University Hospital, Seoul, South Korea; ^2^Department of Medicine, Seoul National University College of Medicine, Seoul, South Korea; ^3^Department of Neurology, Mayo Clinic, Rochester, MN, United States; ^4^Department of Neurosurgery, Mayo Clinic, Rochester, MN, United States; ^5^Department of Psychiatry, Institute of Human Behavioral Medicine in SNU-MRC, Seoul National University College of Medicine, Seoul, South Korea

**Keywords:** amygdala, resting-state functional connectivity, hemispheric asymmetry, insula, social anxiety disorder

## Abstract

**Background:** The amygdala plays a key role in emotional hyperreactivity in response to social threat in patients with social anxiety disorder (SAD). We investigated resting-state functional connectivity (rs-FCN) of the left and right amygdala with various brain regions and functional lateralization in patients with SAD.

**Methods:** A total of 36 patients with SAD and 42 matched healthy controls underwent functional magnetic resonance imaging (fMRI) at rest. Using the left and right amygdala as seed regions, we compared the strength of the rs-FCN in the patient and control groups. Furthermore, we investigated group differences in the hemispheric asymmetry of the functional connectivity maps of the left and right amygdala.

**Results:** Compared with healthy controls, the rs-FCN between the left amygdala and the dorsolateral prefrontal cortex was reduced in patients with SAD, whereas left amygdala connectivity with the fusiform gyrus, anterior insula, supramarginal gyrus, and precuneus was increased or positively deflected in the patient group. Additionally, the strength rs-FCN between the left amygdala and anterior insula was positively associated with the severity of the fear of negative evaluation in patients with SAD (*r* = 0.338, *p* = 0.044). The rs-FCN between the right amygdala and medial frontal gyrus was decreased in patients with SAD compared with healthy controls, whereas connectivity with the parahippocampal gyrus was greater in the patient group than in the control group. The hemispheric asymmetry patterns in the anterior insula, intraparietal sulcus (IPS), and inferior frontal gyrus of the patient group were opposite those of the control group, and functional lateralization of the connectivity between the amygdala and the IPS was associated with the severity of social anxiety symptoms (*r* = 0.365, *p* = 0.037).

**Conclusion:** Our findings suggest that in addition to impaired fronto-amygdala communication, the functional lateralization of amygdala function plays a central role in the pathophysiology of SAD.

## Introduction

Social anxiety disorder (SAD) is characterized by abnormal fear in one or more social situations causing considerable distress, extensive disability, and reduced quality of life (1). Functional neuroimaging studies have demonstrated increased activity in the amygdala and insula of patients with SAD (2). The amygdala plays a central role in emotion recognition, which is essential for social interaction and communication (3). Amygdala damage is associated with impaired recognition of social emotions (4) and impairs eye contact during conversations (5). Previous studies have shown that cortical hubs are disrupted in the functional brain networks of patients with SAD (6) and the prefrontal networks of the amygdala and limbic system are altered (7–9). Amygdala dysfunction is thought to underlie the pathogenesis of SAD and cognitive-behavioral therapy has been shown to down-regulate the abnormally high connectivity of the prefrontal-amygdala network (10).

The left and right amygdala seem to have distinct roles in the emotion regulation process, such that the mediofrontal cortical functional connectivities of the left and right amygdala are differentially modulated by harm avoidance and can be a vulnerability marker for sensitivity to stress and pathologic anxiety (11). Furthermore, the left amygdala is reported to be activated more often than the right amygdala in emotional processing, regardless of stimulus type, task instructions, habituation rates, or elaborate processing (12). Wright et al. (13) found that the right amygdala showed greater habituation to emotional stimuli than the left; however, the left amygdala was significantly more activated than the right to the contrast of fear vs. happy emotions, suggesting that the right amygdala is “part of a dynamic emotional stimulus detection system,” whereas the left is specialized for “sustained stimulus evaluation.” However, little is known about the functional asymmetry of the right and left amygdala in SAD.

The hemispheric laterality of the brain function is beneficial to certain cognitive function (14, 15). Moreover, hemispheric differences in amygdala structure and function have shown clinical implications on fear processing (16–18). With such a fact, it may be demonstrated that bilateral asymmetry in the functional network of the left and right amygdala contributes to the pathogenesis of SAD. We investigated the resting-state functional connectivity (rs-FCN) of the left and right amygdala and hemispheric asymmetry in patients with SAD.

## Materials and methods

### Participants and measurements

Participants included 36 patients with SAD and 42 healthy controls recruited from the psychiatric outpatient clinic at Seoul National University Hospital and the community through an advertisement. Participants were screened using self-report questionnaires, including the Liebowitz Social Anxiety Scale (LSAS), a 24-item measure of fear and avoidance experienced in a range of social interaction and performance situations (19); the Social Interaction Anxiety Scale (SIAS), a 20-item questionnaire that measures the level of anxiety in interpersonal interactions (20); the Social Phobia Scale (SPS), which consists of 20 items that measure performance anxiety (20), the brief version of the Fear of Negative Evaluation scale (B-FNE), a 12-item scale measuring the apprehension of negative evaluation by others (21); and the Beck Depression Inventory (BDI), a 21-item measure of depressed mood (22). The inclusion scores were LSAS ≥ 30, SIAS ≥ 34, and/or SPS ≥ 24 for patients, and SIAS < 34, SPS < 24, B-FNE < 48, and BDI < 21 for control subjects, and ≥12 years of education for both groups. A cutoff score of 21 on the BDI was proposed for Korean population (23, 24). The exclusion criteria for both groups included any history of medical (such as cardiac, respiratory, and hematologic diseases), neurological (such as head trauma, seizure, brain tumor, and stroke), or psychiatric illness (other than SAD and related depressive disorder; such as severe suicidal ideation, substance abuse/dependence, major depressive and bipolar disorders, psychosis, and claustrophobia). Patients were diagnosed according to the criteria of the Diagnostic and Statistical Manual of Mental Disorders Fifth Edition (25) through a clinical interview with a psychiatrist (Choi SH). General anxiety symptoms were assessed using the Hamilton Anxiety Scale (HAS), a 14-item measure of psychic and physical anxiety (26). Four patients with SAD also diagnosed with comorbid depressive disorders. Nine patients were taking routine medications mainly with serotonergic antidepressants. Two of the patients were prescribed benzodiazepines and beta-blocker for as-needed medication; however, they did not take on the day of scanning.

### Functional magnetic resonance imaging acquisition and functional localizer run

Functional magnetic resonance imaging (fMRI) was performed on a 3.0-Tesla MR scanner (Magnetom TrioTrim; Siemens Medical Solutions, Erlangen, Germany). Whole-brain echo-planar images (EPI) were acquired (TR = 2,000 ms; TE = 30 ms; flip angle = 80°; 34 slices; 3.4 mm isotropic voxel resolution). Resting-state fMRI was acquired for 5 min. A high-resolution T1-weighted anatomical image was also acquired (flip angle = 9°; 208 slices; 1.0 mm isotropic voxel size).

EPI time series were acquired during a functional localizer run to determine the seed regions in the left and right amygdala. Participants were asked to look at the screen during the sequence of three conditions: a square checkerboard pattern, an angry face, and a neutral face. Each condition appeared for 10 s, with 10 s of fixation between conditions. The sequence was repeated eight times.

### fMRI data preprocessing

Anatomical T1 images were coregistered to the first functional image using a local Pearson correlation cost function (27). Preprocessing of imaging data was performed using Analysis of Functional NeuroImages (AFNI; http://afni.nimh.nih.gov/afni) software (28). The first three volumes from each functional image were removed. We used Physiologic EStimation by Temporal ICA (PESTICA; http://www.nitrc.org/projects/pestica/) to determine and correct for physiological cardiac and respiratory noise in the resting-state fMRI data (29). After removing physiological noise using PESTICA, images were despiked, and then corrected for slice-time acquisition differences and head motion (30). The slice-timing and motion-corrected functional images were acquired using an anatomy-based image correction method known as ANATICOR (31). Hardware artifacts were modeled with one regressor for eroded local white matter signals and one averaged signal from eroded lateral ventricle masks. The regressors of no interest were (1) six parameters obtained by correction of head motion, (2) signal from the eroded large ventricle mask, and (3) signal from a region of the local white matter erosion mask (*r* = 15 mm). The residual time series were smoothed with a heat kernel that resulted in 4-mm full width-at-half-maximum (FWHM) resolution. At the ANATICOR stage, we performed motion censoring for head motion artifacts (32) using estimated translational and rotational displacement with respect to the *x, y*, and *z* axes. The threshold set was an estimated displacement of <0.3 mm for the Euclidean L2 norm of motion displacement between successive time series volumes. Head-coil artifacts were checked visually by observing the contribution of local white matter signals in the time series, and no strong artifact was detected across brain tissue types (31). Following these quality control steps, data from all subjects met our criteria and were included in this study (32, 33). All imaging data were then transformed to the N27 template space (34).

### Functional connectivity of the amygdala seed locations and hemispheric asymmetry in the functional connectivity maps of the left and right amygdala

Using the amygdala anatomical masks from the FreeSurfer parcellation of the N27 brain template (35), we identified seed regions in the left [Talairach coordinates: (*x* = −17 mm, *y* = −7 mm, *z* = −8 mm)] and right [(19, −5, −10)] amygdala from the contrast map of the angry face vs. the square checkerboard pattern during the localizer run of the control group. For the functional connectivity map of the left amygdala in a single subject, the time series from each voxel within a given seed region of the left amygdala were averaged, a Pearson correlation coefficient was calculated between the averaged time series of the left amygdala and the time series of whole-brain vertices, and then the correlation coefficient was normalized using Fisher's transformation to enable statistical analysis. These procedures were applied to the data of all subjects. A two-sample *t*-test was used to compare the functional connectivity maps of the control and patient groups. The threshold of significance was set at family-wise error (FWE)-corrected *p* < 0.01 (36). The same procedures were used to compare the functional connectivity maps of the right amygdala in the patient and control groups.

To assess between-group differences in the hemispheric asymmetry of connectivity patterns across the left and right seed locations, we flipped the *x*-coordinate values of the individual right amygdala connectivity maps, subtracted those values from their contralateral pairs (left amygdala connectivity maps), and assessed differences using two-sample *t*-tests. The threshold of significance was set at FWE-corrected *p* < 0.01.

To exclude the effect of medication, the same analyses were carried out without nine patients taking medications.

### Statistical analyses

Two-sample *t*-tests and chi-squared tests were used to compare between-group differences in demographic and clinical characteristics. Pearson's correlation coefficients were calculated to assess the relationship between symptom severity and the strength of functional connectivity with the amygdala in patients with SAD. Pearson's correlation analyses were repeated without nine patients taking medications. In addition, we considered the potential influence of comorbid depressed mood by using partial correlation analyses with covariates of the BDI scores in the patient group. Hemispheric asymmetry of left and right amygdala rs-FCN was calculated as the difference in rs-FCN strength of the left vs. right amygdala. All statistical tests were two-tailed with a significance level of 0.05. Additionally, adjusted *p*-values for multiple correlations using a sequential Holm-Bonferroni procedure were considered.

## Results

### Participant demographic and clinical characteristics

The demographic and clinical characteristics of the participants are shown in Table 1. Age, gender, and handedness were not significantly different between groups. The HAS, BDI, and social anxiety scores were higher in the patients than in the control group.

**Table 1:** Participant demographic and clinical characteristics.

**Characteristics**	**Social anxiety disorder (*n* = 36)**	**Control (*n* = 42)**	**χ^2^ or *t***	***p*-value**
Male, *n* (%)	17 (47.2)	19 (45.2)	0.031	0.861
Right/ambidextrous handedness, *n*	33/3	39/3	0.054	0.816
	**Mean (SD; range)**	**Mean (SD; range)**		
Age, in years	25.4 (3.1; 20–33)	24.7 (3.1; 19–32)	1.054	0.295
Educational level, in years	15.6 (2.3; 12–23)	15.6 (1.5; 13–19)^a^	0.132	0.895
Onset age, in years	16.1 (5.1; 8–28)	–	–	–
Global Assessment of Functioning, 0–100	71.7 (9.6; 50–85)	–	–	–
Liebowitz Social Anxiety Scale, 0–144	78.3 (26.2; 35–126)	18.0 (7.7; 1–33)	13.338	< 0.001
Social Interaction Anxiety Scale, 0–80	54.0 (14.8; 24–77)	12.7 (6.5; 2–26)	15.536	< 0.001
Social Phobia Scale, 0–80	39.8 (19.6; 6–65)	4.9 (5.0; 0–24)	10.409	< 0.001
Brief Fear of Negative Evaluation Scale, 12–60	47.9 (9.7; 29–60)	26.4 (6.5; 14–41)	11.360	< 0.001
Hamilton Anxiety Scale, 0–56	28.9 (9.9; 6–47)	6.6 (5.1; 0–19)	11.608	< 0.001
Beck Depression Inventory, 0–63	17.0 (10.4; 2–37)	4.6 (5.5; 0–20)	6.402	< 0.001

a *Data from one participant was missing*.

### rs-FCN of the left and right amygdala

Table 2 shows group differences in rs-FCNs of the left and right amygdala. The right dorsolateral prefrontal cortex was negatively connected with the left amygdala in both groups; however, the patient group showed less negative connectivity than the control group. In contrast, the left and right fusiform gyri were positively connected with the left amygdala in both groups; however, the rs-FCN was greater in the patients than in the control subjects. The rs-FCNs of the left amygdala with the right anterior insula, right supramarginal gyrus, left precuneus, and left cerebellum were positive in the patient group and negative in the control group.

**Table 2:** Group differences in the resting-state functional connectivity of the left and right amygdala.

**Brain region, Brodmann area**	**Talairach coordinates**			**Voxels, *n***	**Max intensity**
	**x**	**y**	**z**		
**LEFT AMYGDALA**					
***|CON|** > **|SAD|***					
R dorsolateral prefrontal cortex, 46/10^a^	33	40	−16	12,853	−4.343
R anterior insula, 13^b^	37	13	0	2,078	−3.940
R supramarginal gyrus, 40^b^	35	−37	33	11,607	−4.500
L cerebellum^b^	−25	−54	−25	2,036	−4.200
***|CON|**<**|SAD|***					
B fusiform gyrus, 20/37^c^	−37	−41	−20	2,798	−4.201
	50	−55	−13	1,309	−3.524
L precuneus, 31/7^b^	−21	−55	29	2,317	−4.420
**RIGHT AMYGDALA**					
***|CON|** > **|SAD|***					
R medial frontal gyrus, 8^c^	7	35	39	3,499	3.647
R middle temporal gyrus, 21^d^	54	−5	−24	3,049	4.285
L supplementary motor area, 6^d^	−1	−21	64	2,136	3.593
	−11	−19	46	1,862	3.269
***|CON|**<**|SAD|***					
L parahippocampal gyrus, 27^c^	−10	−35	−3	1,508	−4.180
L superior temporal gyrus, 22^b^	−40	−25	−8	1,500	−4.289
L lentiform nucleus^d^	−27	−17	11	1,317	3.891

*CON, healthy controls; SAD, social anxiety disorder; B, Bilateral; R, Right; L, Left*.

a *Negative connectivities in both groups*.

b *Negative connectivity in the control group and positive connectivity in the patient group*.

c *Positive connectivities in both groups*.

d *Positive connectivity in the control group and negative connectivity in the patient group*.

The middle frontal gyrus was positively connected with the right amygdala in both groups, although the strength of rs-FCN was reduced in the patient group compared with the control group. The right middle temporal gyrus, bilateral supplementary motor areas (SMA), and left lentiform nuclei were positively connected with the right amygdala in the control group, but negatively connected in the patient group. The positive connection between the right parahippocampal gyrus and right amygdala was greater in the patient group than in the control group. The left superior temporal gyrus showed negative connectivity with the right amygdala in the control group and positive connectivity in the patient group (Table 2).

The additional analyses for participants without medications revealed consistent results (Supplementary Table 1 in Supplementary Material).

### Hemispheric asymmetry of rs-FCN of the left and right amygdala

**Figure 1:**
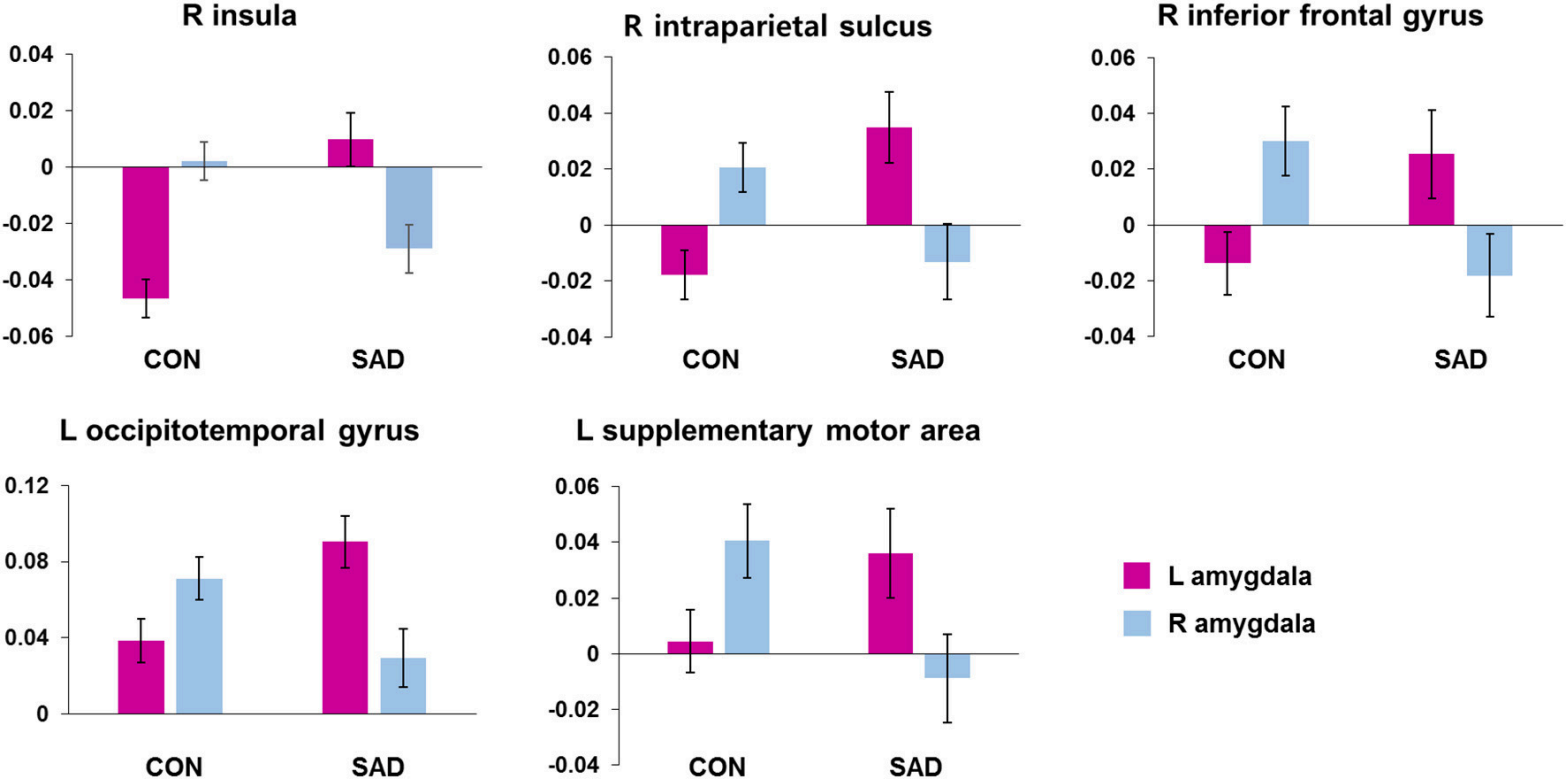
Brain regions showing between-group differences in hemispheric asymmetry in the resting-state functional connectivity of the left and right amygdala. The bar indicates mean ± standard error of *z*-scores of the functional connectivity in each cluster with the Left or Right amygdala. CON, healthy controls; SAD, social anxiety disorder; L, left; R, right.

We found several between-group differences in the hemispheric asymmetry of rs-FCN of the left and right amygdala (Figure 1). Left amygdala rs-FCNs with the right insula [(29, 21, 1), 16,194 voxels], right intraparietal sulcus [IPS, (32, −55, 42), 7,202 voxels], right inferior frontal gyrus [(54, 0, 23), 1,316 voxels], left occipitotemporal cortex [(−41, −65, 0), 4,138 voxels], and left SMA [(−13, −15, 62), 1,412 voxels] exhibited positive connections in the patient compared to negative connections in the control group. Furthermore, the rs-FCN patterns of the left and right amygdala with the insula, IPS, and inferior frontal gyrus in the patient group were opposite from those in the control group. The rs-FCNs of the left amygdala with the occipitotemporal cortex and SMA were dominant in the patient group, whereas the right amygdala was dominant in the control group. The results of additional analyses for participants without medications were presented in Supplementary Material (Supplementary Figure 1).

### Correlations between symptom severity and rs-FCN strength in the left and right amygdala and hemispheric asymmetry in patients with SAD

**Figure 2:**
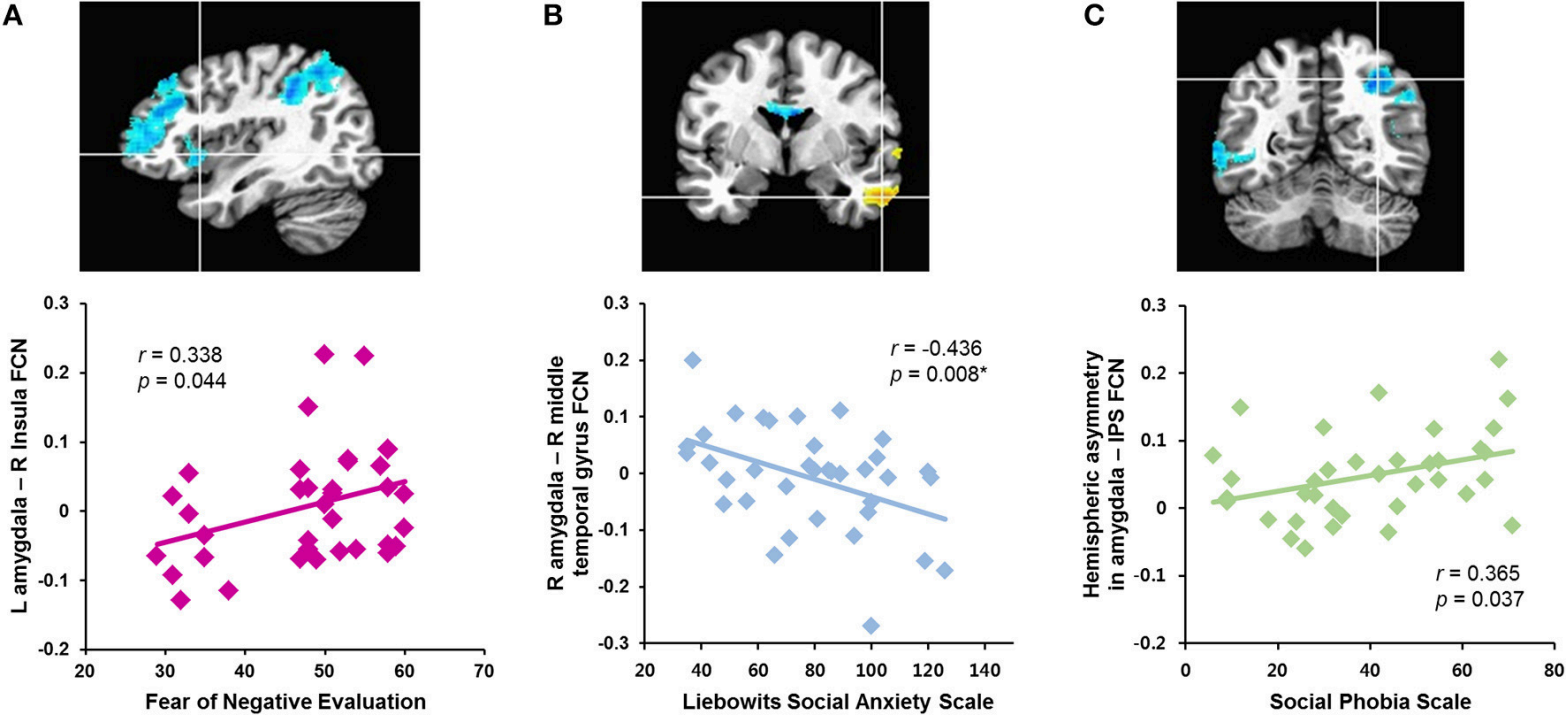
Correlations between symptom severity and rs-FCN strength of brain regions showing between-group differences in the functional connectivity with the left and right amygdala and hemispheric asymmetry in patients with social anxiety disorder. The right insula **(A)**, right middle temporal gyrus **(B)**, right intraparietal sulcus **(C)**. ^*^Significant finding after a sequential Holm-Bonferroni correction for multiple comparisons. L, left; R, right; FCN, functional connectivity; IPS, intraparietal sulcus.

The correlation analyses revealed that the strength of the left amygdala connectivity with the right insula was positively correlated with the fear of negative evaluation (B-FNE score, *r* = 0.338, *n* = 36, *p* = 0.044; Figure 2A). The strengths of the rs-FCNs of the left amygdala with the supramarginal gyrus (*r* = 0.406, *n* = 36, *p* = 0.020) and precuneus (*r* = 0.416, *n* = 36, *p* = 0.017) were correlated with the level of general anxiety (HAS score). None of these correlations were significant after a sequential Holm-Bonferroni correction for multiple comparisons.

The strength of functional connectivity between the right amygdala and middle temporal gyrus was inversely correlated with social anxiety symptom severity (LSAS scores, *r* = −0.436, *n* = 36, *p* = 0.008; SPS score, *r* = −0.439, *n* = 36, *p* = 0.007; Figure 2B). The strength of the rs-FCN between the right amygdala and superior temporal gyrus was positively correlated with level of depressed mood (BDI score, *r* = 0.488, *n* = 36, *p* = 0.003) and general anxiety (HAS score, *r* = 0.446, *n* = 36, *p* = 0.011). All of these correlations remained significant after a correction for multiple comparisons, except for the HAS.

Hemispheric asymmetry in the rs-FCN between the amygdala and the IPS was positively correlated with the social anxiety symptom severity (SPS score, *r* = 0.365, *n* = 36, *p* = 0.037; Figure 2C). This was not significant after a correction for multiple comparisons.

Most of above significant correlation findings were consistent in further analyses for participants without medications and the partial correlation with covariates of the BDI scores (Supplementary Material).

## Discussion

Our findings indicate that communication between the cortical regions of the brain and the left and right amygdala is dysfunctional in patients with SAD, suggesting that the amygdala plays a distinct role in the pathophysiology of SAD. In particular, the positive functional connectivities of the left amygdala with the fusiform face area, insular limbic area, and components of the default mode network (DMN, supramarginal gyrus and precuneus) were greater in patients with SAD than in healthy controls. The insula, IPS, and inferior frontal gyrus showed different patterns of hemispheric asymmetry in rs-FCN of the left and right amygdala in patients with SAD compared with healthy controls. Additionally, our finding that the rs-FCNs between the left and right amygdala and frontal cortices, which play a key role in emotional regulation, were reduced in patients with SAD is consistent with previous studies (2, 37–39).

The fusiform gyrus, a hominoid-specific structure responsible for the ability to recognize faces, is critical for social interaction (40). Accordingly, the fusiform gyrus of patients with SAD has been shown to be pathologically reactive to socially threatening stimuli (41). Interestingly, the rs-FCN between the left amygdala and bilateral fusiform gyri was stronger in patients with SAD than in the healthy control group. Given that the fusiform gyrus is the largest macro-anatomical structure in the ventral temporal cortex and plays an essential role in social emotional processing (42), an exaggerated functional connection between the fusiform gyrus and left amygdala during the resting-state may contribute to the hypervigilant response to facial expressions indicating social threat observed in patients with SAD (42–44).

The anterior insula receives a direct projection from the central nucleus of the amygdala, and the anterior insula itself also projects to the amygdala (45). Emotional induction activates the amygdala, and the insula is involved in emotional processing (46). Given that individuals with major depression exhibit greater activation of the amygdala, insula, and ventrolateral prefrontal cortex in response to increasing social exclusion than controls (47), it may be that abnormal connectivity between the left amygdala and insula in patients with SAD contributes to their vulnerability to pathological social anxiety. A significant association between the fear of negative evaluation and rs-FCN between the left amygdala and insula suggests that this dysfunctional connection underlies the excessive fear of negative evaluation by others experienced by patients with SAD. Moreover, we found that hemispheric asymmetry in rs-FCN of the amygdala in the insula of patients with SAD was dissimilar to that of healthy controls.

Abnormal connectivity between the left amygdala and the precuneus and supramarginal gyrus was associated with the level of general anxiety in the patient group. Given that these regions are part of the DMN (48, 49), which processes introspective thoughts during the resting-state (50), it may be that abnormal positive connectivity between these regions and the left amygdala lead to enhanced emotional surveillance related to self-relevant information. A previous study found negative rs-FCN between the amygdala and precuneus in healthy adults and adolescents and positive rs-FCN between the amygdala and precuneus in adolescents with major depressive disorder (51). Furthermore, the authors reported that the rs-FCN between the left amygdala and precuneus was positively associated with the level of neuroticism in healthy participants. Thus, impaired negative rs-FCN between the left amygdala and DMN regions may underlie the disproportionate “emotional coloring” of self-relevant information processing in patients with SAD (51).

The functional connectivity between the right amygdala and middle temporal gyrus observed in healthy controls was lack in patients with SAD. The robust associations between the disconnection of these regions and symptom severity in the patient group suggest that this abnormality is specific for SAD. Moreover, we previously reported that impaired connectivity of the left middle temporal gyrus was proportional to the severity of functional impairment in patients with SAD (52). Our finding in the present study that the strength of the rs-FCN between the right amygdala and middle temporal gyrus was reduced in patients with SAD provides further evidence that the social-affective communication network is impaired in SAD.

In contrast, the functional connectivities of the right amygdala with the parahippocampal limbic area and superior temporal gyrus were increased in patients with SAD. Enhanced rs-FCN between the superior temporal gyrus and right amygdala was associated with depressed mood and general anxiety. The superior temporal gyrus has been implicated in social-emotional processing, along with other “social brain” regions, including the inferior frontal gyrus, posterior superior temporal sulcus, fusiform gyrus, ventromedial prefrontal cortex, and amygdala (42, 53). Exaggerated functional connectivity within these social brain regions, particularly in the right amygdala, may underlie the emotional susceptibility characteristic of SAD.

It is well known that cognitive capacities, such as language and visuospatial attention, are generally lateralized to one cerebral hemisphere (54, 55). Left-hemisphere regions show a preference for same hemisphere interactions, whereas the right-hemisphere regions interact in a more integrated fashion with both hemispheres. These two patterns of interaction are associated with left-lateralized functions (14). Previous studies have reported functional asymmetry in the processing of emotional stimuli in the bilateral amygdala (13, 56). Given that the left amygdala is associated with sustained stimulus processing, whereas the right amygdala shows greater habituation (13), it is likely that patients with SAD are more affected by left amygdala activity. Our finding that the left amygdala was dominant in rs-FCN with several cortical regions in patients with SAD supports this assumption. In particular, functional asymmetry between the amygdala and IPS, which has been implicated in visual attention and interpreting the intent of others, was correlated with symptom severity in patients with SAD. Given that the amygdala can modulate attention and help direct overt visuospatial attention in face gaze (57), the interconnectivity between the amygdala and IPS may play a critical role in the attentional bias toward negative social cues found in patients with SAD. Thus, increased aberrant connectivities between the left amygdala and the limbic area of the insula and social brain of the IPS and inferior frontal gyrus may underlie pathological social anxiety.

Our study has several limitations. First, as most participants were undergraduate students in their early-to-mid 20s, caution should be exercised in generalizing our findings to SAD patients in a wider age range. Second, there included four patients who have comorbid depressive disorders. Clinically, SAD and depressive symptoms often covary (58). Thus, we included these participants when depressive disorders were judged to have been accompanied by SAD. Additionally, partial correlation analysis with covariates of the level of depressed mood exhibited no significant influence of comorbid depressive symptoms on the original results. Third, there also included ambidextrous participants. Since the ambidextrous participants were < 10% in each group, the results were not considered to be affected significantly. In addition, it was reported that neurophysiological basis of language lateralization is different from that of handedness (59), and arcuate fasciculus asymmetry is independent of hand preference (60). Fourth, we used a liberal significance threshold in the correlation analyses, although a stricter significance threshold was required for individual comparisons to compensate for the number of inferences being made. Thus, the correlation analyses of our study has only an exploratory nature and should be limited in interpretation. However, because the direction of the correlations was consistent with the results of the two-sample *t*-tests of left and right amygdala rs-FCNs, we believe our findings have clinical implications.

In conclusion, we found dissociated functional connections between the left and right amygdala and cortical regions. Increased rs-FCN between the left amygdala and the fusiform face area and reduced rs-FCN between the right amygdala and the middle temporal gyrus were associated with symptom severity of social anxiety in patients with SAD. Moreover, the hemispheric asymmetry of the functional connections of the amygdala with the insula, IPS, and inferior frontal gyrus was altered in patients with SAD. In particular, the abnormal dominant left amygdala functional connectivity with the IPS in patients with SAD was related to symptom severity, which is consistent with the function of these regions in processing visual attention to social threat. We believe the association between hemispheric asymmetry in the functional network of the amygdala and the pathophysiology of anxiety disorders warrants further study in a manner similar to the investigation of hemispheric lateralization of cognitive abilities.

## Ethics statement

This study was approved by the Institutional Review Board of Seoul National University Hospital, and written informed consent was obtained from all participants. Our study was conducted in accordance with the Declaration of the World Medical Association.

## Author contributions

Y-HJ: analyzed the data and wrote the manuscript; JS and YL: contributed to data acquisition and processing; JJ: obtained funding and reviewed and commented on the first draft of the manuscript; HJ: contributed to study concept, formal analysis, study supervision, and writing the manuscript; S-HC: contributed to study concept and design, obtaining funding, and writing the manuscript and has responsibility for conduct of research and final approval.

### Conflict of interest statement

The authors declare that the research was conducted in the absence of any commercial or financial relationships that could be construed as a potential conflict of interest.
