# Supplementary material for: Altered Amygdala Resting-State Functional Connectivity and Hemispheric Asymmetry in Patients With Social Anxiety Disorder
Source: Front Psychiatry. 2018 Apr 26;9:164. doi: 10.3389/fpsyt.2018.00164 (PMC5932339; doi:10.3389/fpsyt.2018.00164)
Supplement: Supplementary file 1 [file Data_Sheet_1.docx]

***Supplementary Material***

**Altered Amygdala Resting-state Functional Connectivity and Hemispheric Asymmetry in Patients with Social Anxiety Disorder**

**Ye-Ha Jung, Jung Eun Shin, Yoonji Lee, Joon Hwan Jang, Hang Joon Jo*, Soo-Hee Choi***

*** Correspondence:** Soo-Hee Choi, soohchoi@snu.ac.kr; Hang Joon Jo, jo.hang@mayo.edu

**1. Supplementary Data**

**Hemispheric Asymmetry of rs-FCN of the Left and Right Amygdala among Participants without Medications**

For participants without medications, several between-group differences in the hemispheric asymmetry of rs-FCN of the left and right amygdala were also found (Figure S1). The rs-FCN patterns of the left and right amygdala with the intraparietal sulcus ([32, -55, 42], 8,096 voxels), supplementary motor area ([-13, -15, 62], 1,193 voxels), medial frontal gyrus ([-9, 30, 50], 1,211 voxels), lateral orbitofrontal cortex ([-34, 30, -6], 1,328 voxels), anterior ([9,26 , 31], 20,912 voxels) and posterior ([4, -42, 27], 2,215 voxels) cingulate cortices, middle temporal gyrus ([-57, -54, -2], 8,510 voxels), and lingual gyrus ([-17, -66, 0], 1,459 voxels) in the patient group were opposite from those in the control group.

**Correlations between Symptom Severity and rs-FCN Strength in the Left and Right Amygdala and Hemispheric Asymmetry in Patients with SAD**

For 27 participants without medications, most of significant correlation findings were still meaningful, except for the positive correlation between the strength of the left amygdala connectivity with the the right insula and the fear of negative evaluation. The strengths of the rs-FCNs of the left amygdala with the supramarginal gyrus (r = 0.454, p = 0.030) and precuneus (r = 0.599, p = 0.003) were correlated with the level of general anxiety (HAS score). The strength of functional connectivity between the right amygdala and middle temporal gyrus was inversely correlated with social anxiety symptom severity (LSAS scores, r = −0.476, p = 0.012; p = 0.040; SPS score, r = -0.561, p = 0.002). The strength of the rs-FCN between the right amygdala and superior temporal gyrus was positively correlated with level of depressed mood (BDI score, r = 0.427, p = 0.026). Hemispheric asymmetry in the rs-FCN between the amygdala and the IPS was positively correlated with the social anxiety symptom severity in a trend level (SPS score, r = 0.358, p = 0.067). The results for between the precuneus and the HAS score and between the middle temporal gyrus and the SPS score were remained significant after a sequential Holm-Bonferroni correction for multiple comparisons.

Partial correlations with covariates of the BDI scores also revealed the consistent results with the original analyses. The strength of the left amygdala connectivity with the right insula was positively correlated with the fear of negative evaluation (B-FNE score, r = 0.498, p = 0.004). The strengths of the rs-FCNs of the left amygdala with the supramarginal gyrus (r = 0.411, p = 0.022) and precuneus (r = 0.393, p = 0.029) were correlated with the level of general anxiety (HAS score). The strength of functional connectivity between the right amygdala and middle temporal gyrus was inversely correlated with social anxiety symptom severity (LSAS scores, r = −0.438, p = 0.014; SPS score, r = -0.414, p = 0.020). The strength of the rs-FCN between the right amygdala and superior temporal gyrus was positively correlated with level of general anxiety (HAS score, r = 0.363, p = 0.045). Hemispheric asymmetry in the rs-FCN between the amygdala and the IPS was positively correlated with the social anxiety symptom severity (SPS score, r = 0.502, p = 0.004). The results for between the insula and the B-FNE score and between the IPS and the SPS score were remained significant after a sequential Holm-Bonferroni correction for multiple comparisons.

**2. Supplementary Table and Figure**

**Supplementary Table 1.** Group differences in the resting-state functional connectivity of the left and right amygdala among participants without medications.

| **Brain region, Brodmann area** | **Talairach Coordinates** | | | **Voxels, n** | **Max intensity** |
| --- | --- | --- | --- | --- | --- |
|  | **x** | **y** | **z** |  |  |
| **Left amygdala** |  |  |  |  |  |
| ***\|CON\| > \|SAD\|*** |  |  |  |  |  |
| R dorsolateral prefrontal cortex, 46/10^a^ | 33 | 40 | 19 | 14,158 | -4.639 |
| R anterior cingulate cortex, 32^a^ | 8 | 25 | 30 | 1,381 | -4.260 |
| R supramarginal gyrus, 40^b^ | 35 | -37 | 33 | 10,431 | -4.672 |
| ***\|CON\| < \|SAD\|*** |  |  |  |  |  |
| B fusiform gyrus, 20/37^c^ | -37 | -40 | -20 | 7,019 | -4.400 |
|  | 50 | -56 | -13 | 2,250 | -4.0185 |
| L hippocampus, 28 ^c^ | -26 | -18 | -8 | 1,412 | -3.296 |
| L lateral orbitofrontal gyrus, 47^c^ | -41 | 22 | -9 | 2,413 | -4.585 |
| L middle frontal gyrus, 10/32^b^ | -23 | 45 | 9 | 1,948 | -3.704 |
| L angular gyrus, 39^b^ | -29 | -59 | 29 | 2,344 | -4.258 |
| L intraparietal sulcus, 7/40^b^ | -29 | -42 | 38 | 2,221 | -3.889 |
| **Right amygdala** |  |  |  |  |  |
| ***\|CON\| > \|SAD\|*** |  |  |  |  |  |
| L medial frontal gyrus, 8^d^ | -9 | 46 | 34 | 2,617 | 3.260 |
| R middle temporal gyrus, 21^c^ | 51 | -6 | -24 | 2,685 | 3.884 |
| L lentiform nucleus^d^ | -27 | -17 | 11 | 2,076 | 3.768 |
| Supplementary motor area, 6^d^ | 0 | -17 | 64 | 1,664 | 3.502 |
| ***\|CON\| < \|SAD\|*** |  |  |  |  |  |
| L parahippocampal gyrus, 27^c^ | -10 | -35 | -3 | 1402 | -3.892 |
| L fusiform gyrus, 20/35^c^ | -31 | -31 | -17 | 3,478 | -4.391 |

CON, healthy controls; SAD, social anxiety disorder; B, Bilateral; R, Right; L, Left.

^a^ Negative connectivities in both groups. ^b^ Negative connectivity in the control group and positive connectivity in the patient group. ^c^ Positive connectivities in both groups. ^d^ Positive connectivity in the control group and negative connectivity in the patient group.


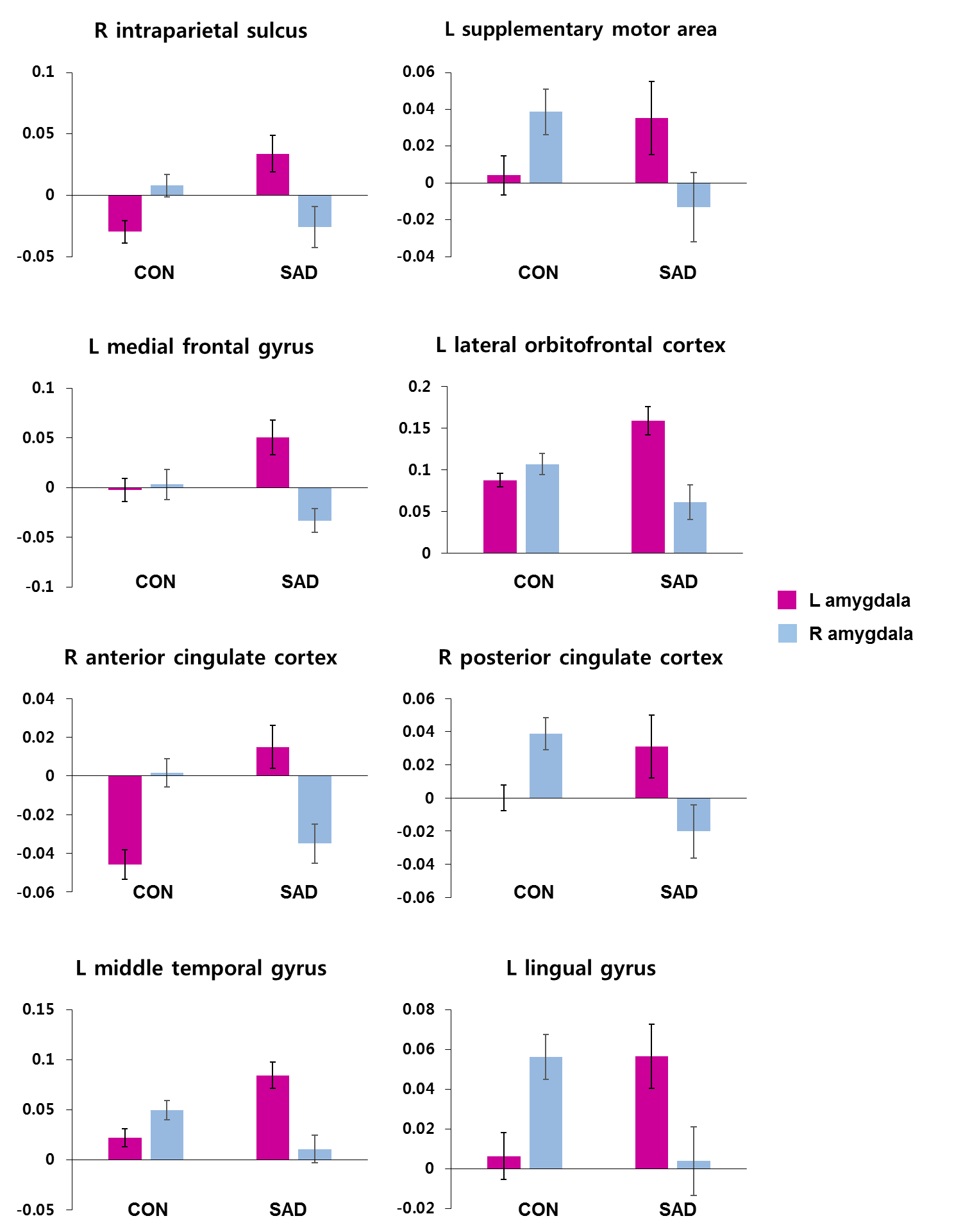


**Supplementary Figure 1.** Brain regions of participants without medications showing between-group differences in hemispheric asymmetry in the resting-state functional connectivity of the left and right amygdala. The bar indicates mean ± standard error of z-scores of the functional connectivity in each cluster with the left or right amygdala. CON, healthy controls; SAD, social anxiety disorder; L, left; R, right.
